# Roles of Palmitoleic Acid and Its Positional Isomers, Hypogeic and Sapienic Acids, in Inflammation, Metabolic Diseases and Cancer

**DOI:** 10.3390/cells11142146

**Published:** 2022-07-08

**Authors:** Miguel A. Bermúdez, Laura Pereira, Cristina Fraile, Laura Valerio, María A. Balboa, Jesús Balsinde

**Affiliations:** 1 Instituto de Biología y Genética Molecular, Consejo Superior de Investigaciones Científicas (CSIC), 47003 Valladolid, Spain; mabermudez@ibgm.uva.es (M.A.B.); lpereira@ibgm.uva.es (L.P.); cristina.fraile.sanchez@alumnos.uva.es (C.F.); laura.valerio@alumnos.uva.es (L.V.); mbalboa@ibgm.uva.es (M.A.B.); 2 Centro de Investigación Biomédica en Red de Diabetes y Enfermedades Metabólicas Asociadas (CIBERDEM), Instituto de Salud Carlos III, 28029 Madrid, Spain

**Keywords:** hexadecenoic fatty acid, diabetes and obesity, cardiovascular disease, liver disease, lipid signaling, lipid compartmentalization

## Abstract

In the last few years, the monounsaturated hexadecenoic fatty acids are being increasingly considered as biomarkers of health with key functions in physiology and pathophysiology. Palmitoleic acid (16:1n-7) and sapienic acid (16:1n-10) are synthesized from palmitic acid by the action of stearoyl-CoA desaturase-1 and fatty acid desaturase 2, respectively. A third positional isomer, hypogeic acid (16:1n-9) is produced from the partial β-oxidation of oleic acid. In this review, we discuss the current knowledge of the effects of palmitoleic acid and, where available, sapienic acid and hypogeic acid, on metabolic diseases such as diabetes, cardiovascular disease, and nonalcoholic fatty liver disease, and cancer. The results have shown diverse effects among studies in cell lines, animal models and humans. Palmitoleic acid was described as a lipokine able to regulate different metabolic processes such as an increase in insulin sensitivity in muscle, β cell proliferation, prevention of endoplasmic reticulum stress and lipogenic activity in white adipocytes. Numerous beneficial effects have been attributed to palmitoleic acid, both in mouse models and in cell lines. However, its role in humans is not fully understood, and is sometimes controversial. Regarding sapienic acid and hypogeic acid, studies on their biological effects are still scarce, but accumulating evidence suggests that they also play important roles in metabolic regulation. The multiplicity of effects reported for palmitoleic acid and the compartmentalized manner in which they often occur, may suggest the overlapping actions of multiple isomers being present at the same or neighboring locations.

## 1. Introduction

The ratio of saturated to monounsaturated fatty acid (MUFA) is an important parameter regulating the fluidity of biological membranes. Deregulation of this ratio due to decreased MUFA levels may be instrumental to the onset of diseases such as diabetes, cardiovascular disease or cancer [1,2]. Thus, MUFA levels are increasingly being considered as health markers. Stearoyl-CoA desaturase (SCD) (Δ9 desaturase) is the rate-limiting enzyme for the biosynthesis of the major MUFA present in cells and tissues, oleic acid (*cis*-9-octadecenoic acid, 18:1n-9) [3]. Thus, pharmacological manipulation of SCD activity has repeatedly been suggested as a strategy to treat metabolic diseases and cancer [4,5,6]. Two SCD isoforms are present in humans, SCD-1 and SCD-5. While the former is ubiquitously expressed, the latter appears to be mainly expressed in brain. In mice, the situation is more complex, as four isoforms have been identified and characterized (SCD-1 to -4) [7].

Another family of MUFAs that stands out for its possible health benefits is that of the hexadecenoic fatty acids (16:1). Palmitoleic acid (*cis*-9-hexadecenoic acid, 16:1n-7) is the most abundant member of the family and, likely because of this, the most studied. This isomer appears to have unique biological actions in modulating metabolic responses, which has led to the concept of it serving as a lipid hormone, or ‘lipokine’ that coordinates metabolic responses between tissues [8].

Two positional isomers of 16:1n-7 acid with increasing importance in physiology and pathophysiology have recently been described. These are sapienic acid (*cis*-6-hexadecenoic acid, 16:1n-10) and hypogeic acid (*cis*-7-hexadecenoic acid, 16:1n-9) [9,10,11]. Traditionally described as a component of human sebum, hair, and nails, 16:1n-10 has also recently been found in many other cells, including human red blood cells and cells of the innate immune system [10,11,12]. 16:1n-9 was found at significant levels in the neutral lipid fraction of foamy monocytes, and was later identified in several cells of human and murine origin [13,14,15,16]. For the sake of completion, it is worth noting that at least a fourth hexadecenoic fatty acid isomer is present at measurable levels in mammalian cells, namely palmitvaccenic acid (*cis*-11-hexadecenoic acid, 16:1n-5) [10]. As no specific role or functional involvement has been described for this isomer yet, it will no longer be considered in this review.

**Figure 1:**
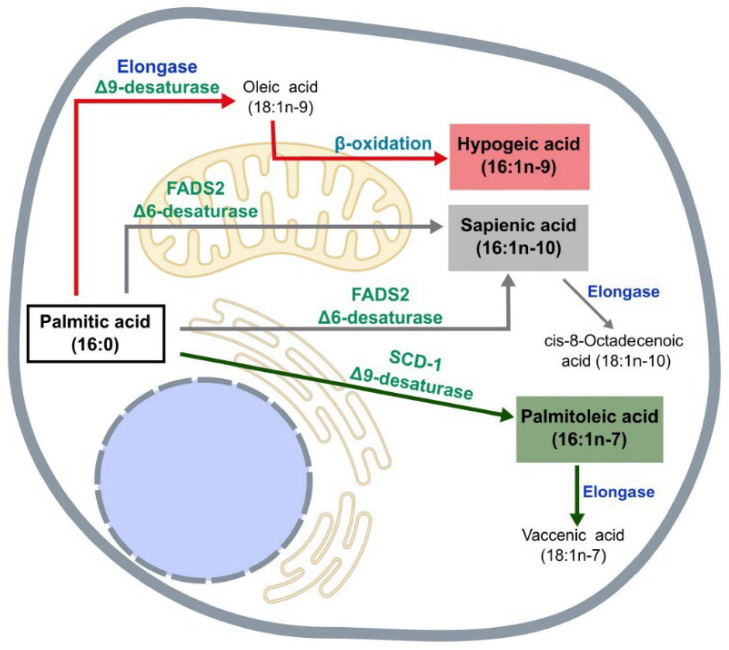
Pathways of biosynthesis of hexadecenoic fatty acids. Palmitoleic acid (16:1n-7) is synthesized in the endoplasmic reticulum via desaturation of palmitic acid (16:0) at C9 via SCD. Hypogeic acid (16:1n-9) is synthesized in the mitochondria via β-oxidation of oleic acid (18:1n-1). Sapienic acid (16:1n-10) is synthesized in the mitochondria and endoplasmic reticulum via desaturation of 16:0 at C6 via FADS2.

The biochemical pathways for the synthesis of the three major hexadecenoic fatty acid positional isomers are shown in Figure 1. 16:1n-7 is synthesized directly from palmitic acid by the enzyme SCD-1. In humans, 16:1n-7 biosynthesis occurs primarily in liver and to a lesser extent in adipose tissue, where it is later incorporated into phospholipids, triacylglycerol, and cholesterol esters. Its subsequent elongation gives rise to *cis*-vaccenic acid (18:1n-7). 16:1n-9 is generated from the partial β-oxidation of oleic acid. The latter usually comes from the diet, but it may also be synthesized from palmitic acid by elongation and subsequent desaturation by SCD-1 (Figure 1). Finally, 16:1n-10 is formed by the desaturation of palmitic acid at C6 by the enzyme fatty acid desaturase (FADS) 2. In turn, 16:1n-10 can be elongated to form *cis*-8-octadecenoic acid (18:1n-10).

Humans express three FADS genes, named FADS1, FADS2, and FADS3. FADS1 is known as a Δ5 desaturase, FADS2 is generally known as a Δ6 desaturase, and FADS3 as a ceramide desaturase [17]. Palmitic acid and the polyunsaturated fatty acids of the n-3 and n-6 series are competitors for the Δ6 desaturase reaction, which may limit the amount of 16:1n-10 produced by some cells [10,18]. It is striking that, while SCD-1-mediated Δ9 desaturation (leading to 16:1n-7 formation) takes place in the endoplasmic reticulum, FADS2-mediated Δ6 desaturation (leading to 16:1n-10 formation) also occurs in the mitochondria [19,20]. The later organelle is also the site of synthesis of 16:1n-9 via β-oxidation [9] (Figure 1). Clearly, compartmentalization of lipid synthesis, turnover and signaling must play a paramount role in regulating the metabolic and inflammatory reactions of cells and tissues [19,20,21]. In particular, the partition of palmitic acid between Δ9 (synthesis of palmitoleic acid) and Δ6 desaturase pathways (synthesis of sapienic acid) is beginning to be considered as a metabolic switch in health and disease [22,23].

In this review, we discuss the involvement and effects of 16:1n-7 on chronic metabolic diseases such as obesity, diabetes, cardiovascular diseases, atherosclerosis, and nonalcoholic fatty liver disease (NAFLD), and cancer. When data are available, functional roles of the isomers 16:1n-10 and 16:1n-9 are considered as well. Given the anti-inflammatory and metabolic properties recently described for these two isomers, it is possible that the multiplicity of effects and marked compartmentalization of the 16:1n-7 effects could be due, at least in part, to the overlapping actions of hexadecenoic fatty acid isomers being present at the same or neighboring locations [24,25,26,27].

## 2. Obesity and Diabetes

Obesity is one of the major risk factors for the development of diabetes and associated metabolic disorders. The excessive increase in fat deposits in the body leads to a dysregulation of adipose tissue function. This impairment may lead to an increased release and concentration of free fatty acids, glycerol, hormones and inflammatory cytokines in the circulation. All of these alterations have been associated with different health problems such as dyslipidemia, hypertension and insulin resistance, collectively referred to as “metabolic syndrome” [28].

The first evidence of a specific role for 16:1n-7 in metabolic syndrome was provided by Cao et al. [8]. Release of this fatty acid from the adipose tissue acted to suppress steatosis in the liver and improve insulin signaling in muscle. Overall, the study suggested that 16:1n-7 exerts anti-inflammatory effects in the adipose tissue of mice that help mitigate the impact of obesity. Thus, 16:1n-7, but not palmitic acid, suppressed cytokine expression in adipocytes but not in stromal vascular cells, pointing to the former cells as the major target for 16:1n-7 [8]. The authors proposed that 16:1n-7 acted as a lipokine on the basis of: (i) its specific behavior during de novo lipogenesis, accumulating in adipose tissue instead of being a simple intermediate, and (ii) its extremely rapid fluctuation as a reflection of this lipogenesis [8].

Studies utilizing high fat diet (HFD)-fed mice revealed that 16:1n-7 administration improves the whole-body insulin sensitivity and glucose uptake into adipose tissue through the regulation of GLUT-4 and AMPK phosphorylation [29]. 16:1n-7 increased lipolysis and enhanced the expression levels of Atgl and Hsl in adipocytes by a mechanism requiring a functional PPARα [30]. These results unveiled the importance of a fully functional lipid sensor PPARα for 16:1n-7 to exert its positive effects on lipolysis and lipase expression. Further studies also indicated that 16:1n-7 treatment increases oxygen consumption, fatty acid oxidation, and ATP content in white adipocytes [31]. In other studies it was also found that 16:1n-7 treatment prevented the increase of the transcription factors CEBPα and PPARγ in subcutaneous inguinal adipocytes in HFD-treated mice. In addition, 16:1n-7 partially reversed the expression levels of key metabolic target genes of these transcriptional factors involved in glucose and fatty acid uptake (Lpl, Fabp4, and Glut-4), lipogenesis (Fasn, Acc1, Lpin, Dgat1, and Dgat2), and lipolysis (Atgl, and Hsl) [32]. Similar data were reported in a study administering macadamia nut oil (where 16:1n-7 represents 20% of total fatty acid) to HFD-fed mice [33]. Overall, these studies suggest that the beneficial metabolic effects of 16:1n-7 in obesity are brought at least in part, by prevention of gene expression on adipocytes.

On the other hand, studies in humans have often produced different outcomes [25,26,27]. Epidemiological and diet-intervention studies have suggested a positive correlation between obesity and high serum levels of 16:1n-7 [24,34,35], and another study with healthy young Canadians found a positive correlation between the circulating levels of 16:1n-7 and inflammation markers [36]. In agreement with these data, a European dietary intervention study also found an association between obesity and high concentrations of 16:1n-7 [37,38], casting doubts on any potential benefit of the fatty acid [39]. It should be kept in mind when analyzing human data that, in many of the studies, elevated levels of saturated palmitic acid constitute a major cause for the 16:1n-7 elevations, as the former is the direct precursor of the latter. Thus any potential positive effect of 16:1n-7 could be masked by the presence of high levels of saturated fatty acids, which are well known to correlate with adverse health outcomes. This differs from the studies with mice, where 16:1n-7, not palmitic acid, is administered, thus allowing a direct examination of the effects of the MUFA in the absence of elevated levels of saturated fatty acids. 

Some studies have suggested that high levels of 16:1n-7 in red blood cell membranes may constitute a risk factor for the development of obesity-related disorders such as metabolic syndrome [40] and type-2 diabetes [41]. However, other studies found no association between 16:1n-7 levels in plasma and erythrocyte membranes with obesity, insulin resistance or cardiovascular disease [42,43]. It is important to note in this regard that the concentration of a given fatty acid in the membrane of red blood cells does not necessarily have to correspond with the concentration of said fatty acid in plasma or tissues, as in the latter many multiple factors may be involved [44]. Interestingly, recent studies have detected elevated amounts of 16:1n-10 in the membranes of circulating red blood cells from morbidly obese individuals but not in the neutral lipid fraction (cholesterol esters and triacylglycerol). This was in contrast to 16:1n-7 levels, which increased in both membrane and neutral lipid fractions [12].

In vitro studies in a number of cell types to which 16:1n-7 was exogenously applied have generally suggested a number of benefits on insulin signaling and insulin action. For example, treatment of isolated rat pancreatic islets with 16:1n-7 has long been shown to increase insulin secretion, and protect and maintain the proliferation rate of β-cells from death promoted by high levels of glucose or palmitic acid [45,46]. An increase in insulin uptake was also described in rat muscle cells, where the increased expression of the glucose transporter genes Glut-1 and Glut-4 was also noted [47]. Importantly, the addition of insulin after treating the cells with 16:1n-7 produced little or no change to the effects of the fatty acid, suggesting a similar mechanism of action for both 16:1n-7 and insulin [47].

In mouse models of obesity-generated type-2 diabetes, administration of 16:1n-7 was found to reduce insulin resistance and dyslipidemia [48], and similar results were reported in a study utilizing obese sheep [49]. Experiments in rat models of metabolic syndrome, diet supplementation with macadamia oil resulted in a decrease in fasting plasma insulin and a significant reduction in visceral fat [50]. However, another study utilizing mice fed a hyperlipidemic diet plus the macadamia oil supplement did not detect any reduction in the high glucose levels of these animals [51].

**Figure 2:**
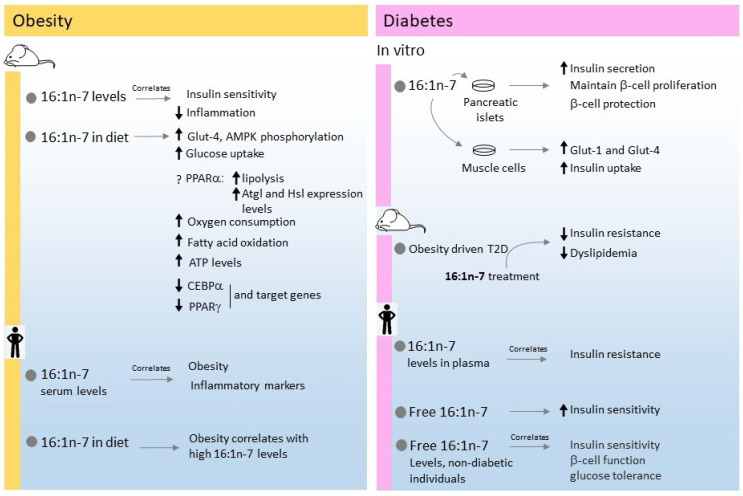
Summary of the effects of 16:1n-7 on metabolic and inflammatory conditions in obesity and diabetes.

Studies on clinical uses of 16:1n-7 in humans for the treatment of type-2 diabetes are scarce. Most of the studies focus on establishing relationships between different parameters related to type 2 diabetes risk and 16:1n-7 plasma or serum concentrations [52]. A positive association between high plasma 16:1n-7 levels and insulin resistance was described in a study of 1800 participants. Moreover, this high fatty acid content was related to a high intake of carbohydrates, alcohol, protein, and high body mass index [53]. Two other studies showed a strong association between high levels of 16:1n-7 in red blood cell membranes and plasma lipids (cholesterol esters, triacylglycerol and phospholipids) with an increased incidence of the diagnosis of type 2 diabetes in the following 5 years [54,55]. While these studies focused on esterified levels of 16:1n-7, other studies have found a strong correlation between 16:1n-7 in free fatty acid form (but not total fatty acid amount) and improved insulin sensitivity independent of sex, age and adiposity in about 100 participants at increased risk for type 2 diabetes [56]. In addition, a recent longitudinal analysis, conducted in about 1000 non-diabetic patients, showed that plasma free 16:1n-7 can be regarded as an independent determinant of insulin sensitivity, β-cell function and glucose tolerance in non-diabetic individuals [57]. Figure 2 summarizes the studies with 16:1n-7 described in this section.

Regarding the role of other 16:1 isomers in diabetes, we are only aware of the study of Bukowiecka-Matusiak et al. [58], who described a remarkable increase in 16:1n-10 levels in the erythrocyte membranes of women diagnosed with gestational diabetes.

## 3. Cardiovascular Disease and Atherosclerosis

The term cardiovascular disease (CVD) refers to chronic diseases of the heart and circulatory system and is the major cause of mortality in developed countries. It is estimated that by the year 2030, 23.6 million people will die of CVD per year. The risks for CVD are well-known: high serum LDL-cholesterol concentration, high blood pressure, obesity, diabetes, male gender and physical inactivity [59].

Currently, studies addressing the effects of 16:1n-7 on CVD remain inconclusive. In a cardiovascular health study (CHS) cohort, 16:1n-7-containing phospholipid levels in plasma have been associated with an increase in HDL-cholesterol, a reduction in LDL-cholesterol, but enhanced triacylglycerol levels [35,53]. Studies using diets supplemented with 16:1n-7-rich macadamia oil have shown decreases in total plasma cholesterol, LDL-cholesterol, and body weight, an increase in HDL-cholesterol levels, and no changes in triacylglycerol [60,61]. Conversely, elevated 16:1n-7 levels in serum have been associated in other studies with different cardiovascular risk factors such as high triacylglycerol, apoA-1, apoB, blood pressure and endothelial malfunctions [62,63]. Positive associations between red blood cell membrane 16:1n-7 levels and coronary heart disease risk have also been reported in another study, albeit in this case the levels of the 16:1n-7 elongation product vaccenic acid (18:1n-7) in red blood cells inversely correlated with disease risk [64].

Atherosclerosis is a major cause of cardiovascular disease and one of the leading causes of death in developed countries. The atherosclerotic process has been described as an inflammatory disorder, with clear similarities to other pathologies such as diabetes [65]. The initial step of atherosclerosis involves the glycation and oxidation of circulating apoB-containing lipoproteins and their accumulation in the intima. The latter process constitutes a danger signal for the endothelial cells, which recruit circulating monocytes by increasing the expression of the adhesion molecules ICAM/VCAM [66,67,68]. Saturated fatty acids such as palmitic and stearic acids have been found to enhance the monocyte recruitment, as they further enhance the expression of surface adhesion molecules and also promote the release of pro-inflammatory cytokines such as tumor necrosis factor-α and interleukin-6 [27].

**Figure 3:**
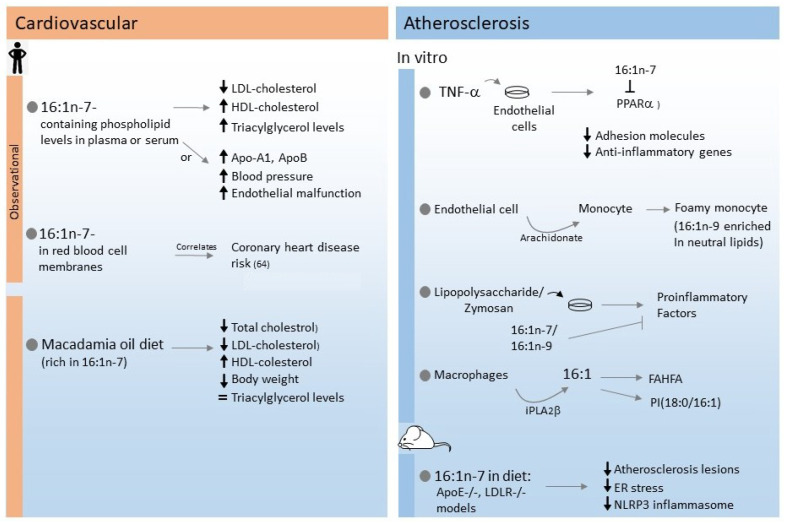
Summary of effects of 16:1n-7 on metabolic and inflammatory conditions in cardiovascular disease and atherosclerosis.

16:1n-7 has been shown to reduce the surface expression of adhesion molecules and the induction of proinflammatory genes in TNFα-stimulated endothelial cells. These effects were related to the inhibition of PPARα expression by the fatty acid [69]. Studies with ApoE^−/−^ and LDLR^−/−^ mice indicated that supplementation of their diet with 16:1n-7 helps reduce the atherosclerotic lesions [70]. These data are in agreement with other studies which, in addition, indicated that the 16:1n-7 effects work through reduction of endoplasmic reticulum stress and activation of the NLRP3 inflammasome [71]. Interestingly, these effects of 16:1n-7 were not reproduced by its trans isomer, suggesting that the double bond configuration is critical for atheroprotection [72]. Figure 3 summarizes the results discussed in this section.

In atherosclerosis lesions, activated endothelial cells release proinflammatory mediators that promote the transformation of the recruited monocytes into foam cells prior to their extravasation and further differentiation to foam macrophages in the intima [73,74,75]. Studies of the inflammatory state in early atherosclerotic plaques have shown that arachidonic acid is released in significant amounts to the bloodstream. Several processes contribute to this release, including endothelial cells interacting with modified lipoproteins, platelets recruited to the activated endothelium, and secreted phospholipase A_2_s acting in situ on circulating lipoproteins [75,76,77,78]. The released arachidonate contributes to lipid droplet formation in the circulating monocytes, thus favoring their transformation into foamy, pro-atherogenic monocytes [66,79,80]. Lipidomic analyses of the composition of these foamy human monocytes demonstrated the selective enrichment of the neutral lipid fraction with 16:1n-9, otherwise an uncommon fatty acid [9,13,81]. The selective accumulation of an uncommon fatty acid such as 16:1n-9 in the neutral lipids of phagocytic cells could represent a warning sign of atherogenicity and, therefore, a possible biomarker of early cardiovascular damage. In this regard, it is interesting to note a recent report showing that chronic supplementation of mice with arachidonate also results in these animals exhibiting elevated 16:1n-9 levels [82]. Another recent study found an inverse association between the levels of nonesterified 16:1n-9 levels in serum and incident stroke, which raises the possibility that this fatty acid could also be considered a potential biomarker for incident stroke risk [16].

**Figure 4:**
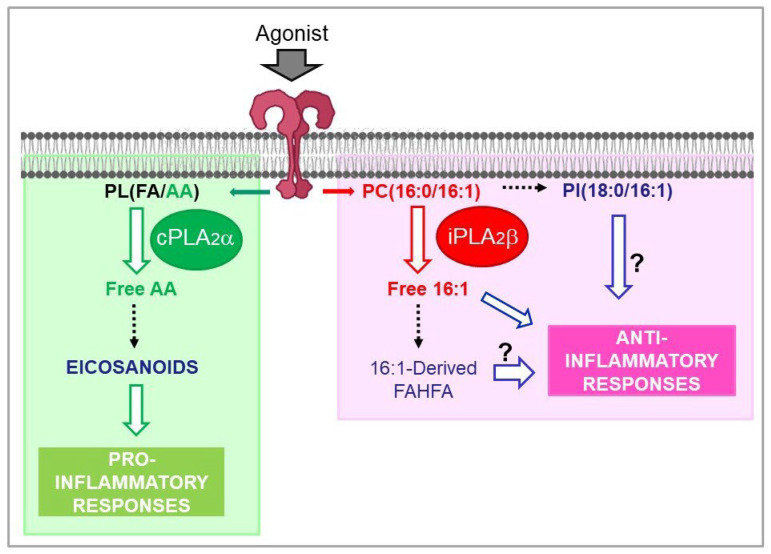
Phospholipase A_2_-mediated signaling in activated macrophages. The macrophages utilize two cytosolic phospholipase A_2_s of similar size but with clearly differentiated functions, to effect lipid signaling leading to either pro- or anti-inflammatory actions. On the one hand, group IVA calcium-dependent phospholipase A_2_ (cPLA_2_α) releases arachidonic acid (AA) from membrane phospholipids, giving rise to the formation of pro-inflammatory eicosanoids. On the other hand, group VIA calcium-independent phospholipase A_2_ (iPLA_2_β) controls the metabolism of hexadecenoic fatty acids (palmitoleic acid, hypogeic acid, and sapienic acid), thus regulating new lipid metabolic pathways with anti-inflammatory character. The abbreviation PL(FA/AA) represents any given phospholipid (PL) containing an unspecified fatty acid (FA) at the sn-1 position, and arachidonic acid (AA) at the sn-2 position. PC(16:0/16:1), 1-palmitoyl-2-hexadecenoyl-*sn*-glycero-3-phosphocholine; PI(18:0/16:1), 1-stearoyl-2-hexadecenoyl-*sn*-glycero-3-phospho-inositol; FAHFA, branched fatty acyl esters of hydroxy fatty acids.

Exogenous administration of both 16:1n-7 and 16:1n-9 to animal models or to cell cultures has shown that both fatty acids manifest a marked anti-inflammatory character, being able to counteract the effects of a number of pro-inflammatory agonists such as bacterial lipopolysaccharide and yeast-derived zymosan particles [9,10,69,83]. While the molecular mechanisms involved are yet to be fully established, a major issue in this area is to define the mechanisms of regulation of 16:1 fatty acid levels and the pathways for incorporation and remodeling of the different lipid pools that contain them. Studies in macrophages have demonstrated that both 16:1n-7 and 16:1n-9 manifest a very strong propensity to incorporate into one single phospholipid species, namely PC(16:0/16:1) (1-palmitoyl-2-hexadecenoyl-*sn*-glycero-3-phosphocholine), which constitutes more than 80% of total incorporated fatty acid [10,84]. Upon cell activation both 16:1n-7 and 16:1n-9 are selectively mobilized from its phospholipid storage site by the action of group VIA calcium-independent phospholipase A_2_, or iPLA_2_β [84]. Of note, this phospholipase A_2_ is a different form from the one that effects arachidonic acid mobilization for eicosanoid biosynthesis, i.e., the group IVA calcium-dependent cytosolic enzyme, or cPLA_2_α [85,86]. Thus, inflammatory activation of macrophages activates parallel but independent metabolic pathways that result in the production of bioactive lipids under separate mechanisms of regulation [86,87] (Figure 4). 

Due to the marked anti-inflammatory character of both 16:1n-7 and 16:1n-9, it is conceivable that the release of these two fatty acids constitutes an important regulatory step for the initiation of pathways aimed at counteracting inflammatory damage [86,87]. These may include formation of 16:1-containing branched fatty acid esters of hydroxy fatty acids (FAHFA), a novel class of bioactive lipids with anti-inflammatory properties [88]. Part of the 16:1 fatty acids released by the activated macrophages have indeed been found to be used for FAHFA synthesis [84]. Importantly, levels of the circulating 16:1n-7 ester of 9-hydroxystearic acid correlate with protective cardiovascular biomarkers in healthy humans [89] (Figure 4).

Finally, a significant part of the 16:1 fatty acids released by macrophages are incorporated into another phospholipid species, namely PI(18:0/16:1) (1-stearoyl-2-hexadecenoyl-*sn*-glycero-3-phosphoinositol) [86]. In analogy with other inositol phospholipid species that are formed upon cell activation and exert defined biological functions [90,91,92,93], it seems likely that the regulated formation of PI(18:0/16:1) may also be physiologically or pathophysiologically relevant (Figure 4).

## 4. Non Alcoholic Fatty Liver Disease (NAFLD) and Non Alcoholic Steatohepatitis (NASH)

NAFLD and NASH have attracted much attention due to their increasing prevalence in Western countries [94,95]. Both diseases share common features such as hepatic lipotoxicity associated with a low-grade chronic inflammation. It remains a great challenge to obtain an unambiguous diagnosis before the disease progresses to fibrosis [94,95]. An early feature of the disease is the condition known as fatty liver, which arises as a consequence of distorted triacylglycerol metabolism [96,97,98].

The biological role of 16:1n-7 in liver disease has been profusely studied, and the results have often been contradictory. The use of mouse models of liver disease has generally suggested that 16:1n-7 exerts protective effects on NAFLD progression. Thus, chronic administration of 16:1n-7 to mice was found to reduce lipid accumulation and the expression of lipogenic genes (Srebp-1, Fas, and Scd1) in the liver [48]. At a molecular level, these effects were correlated with activation of the PPARα/AMPK pathway, which also increased insulin secretion and improved glucose homeostasis [48]. PPARα is a transcription factor that regulates the storage and mobilization of fatty acids by coordinating the expression of key metabolic regulatory enzymes [99]. Another study employing HFD-fed mice confirmed that the beneficial effects of 16:1n-7 on glucose and lipid homeostasis in the liver were also mediated by PPARα-dependent AMPK activation [100].

In addition to these metabolic regulatory effects of PPARα, activation of this transcription factor is known to inhibit NF-κB activation, thereby reducing the expression of proinflammatory genes and the production of proinflammatory cytokines [101]. However, a number of studies using PPARα k.o. mice have shown that the anti-inflammatory effects of 16:1n-7 in models of NAFLD and NASH do not occur downstream of PPARα activation [102,103,104]. On the other hand, it has been suggested that 16:1n-7 prevents lipoapoptosis in human and murine hepatocytes by reducing endoplasmic reticulum stress, which in turn blunts activation of the downstream death mediator Bax [105]. Very recent work suggests that down-regulation of sirtuin-3 expression by 16:1n-7 may account, at least in part, for the anti-inflammatory actions of the fatty acid [106].

**Figure 5:**
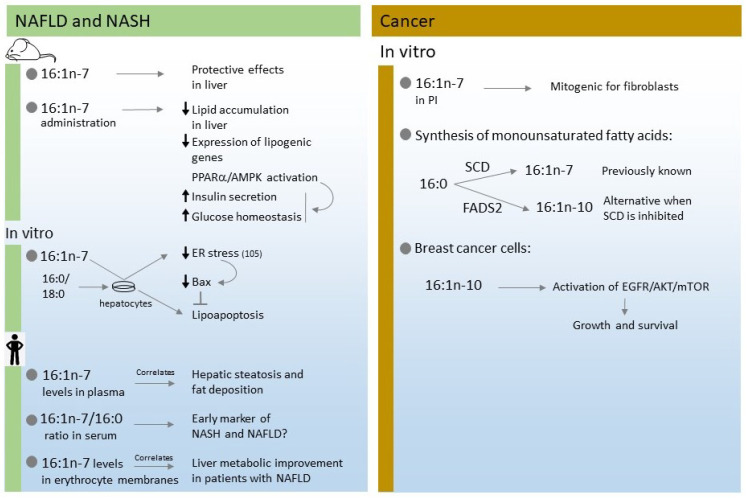
Summary of effects of hexadecenoic fatty acids in liver diseases (**left**) and cancer (**right**).

In humans, circulating levels of 16:1n-7 have been reported to correlate positively with the degree of hepatic steatosis, as well as with fat deposition in hepatocytes [25,42,53]. Indeed, an elevated 16:1n-7/16:0 ratio in serum was proposed as a non-invasive early marker for NASH or NAFLD [94]. The 16:1n-7/16:0 ratio was higher in NASH patients compared to NAFLD patients, and correlated with the hepatic inflammation and fibrosis status [94]. Similarly, a study with 41 Chinese pediatric patients aged 4 to 17 showed that serum 16:1n-7 concentrations were higher in the mild and severe groups of NAFLD compared with the control group [107]. Moreover, a study on the fatty acid profile of erythrocyte membranes of 55 adult patients with NAFLD who were subjected to a 6-month dietary intervention in order to reduce fatty liver, established that reduction of several fatty acids including 16:1n-7, directly correlated with liver metabolic improvement [108]. Figure 5 summarizes the effects of 16:1n-7 on NAFLD and NASH discussed in this section.

## 5. Cancer

Cancer is characterized by the development of abnormal cells that divide uncontrollably and display profound metabolic changes. For instance, lipid metabolism experiences a dramatic shift toward enhancement of lipid biosynthesis pathways [109,110]. Increased lipid uptake, storage, and lipogenesis are strongly up-regulated in tumor cells to maintain the structure and fluidity of the cell membrane [111,112,113]. To sustain these increased rates of fatty acid synthesis, the enzymes that generate MUFA such as FADS2 and SCD1 are frequently overexpressed [113,114]. Selective inhibition of these enzymes has been found to translate into anticancer activity in vitro [115,116]. In lung and prostate cancer cells, SCD1 inhibition decreased the de novo fatty acid synthesis and the MUFA/SFA ratio, thus interrupting cell proliferation [6,115,117,118]. The effects of SCD1 blockade could be reversed by administering a number of MUFAs, including 16:1n-7 [117,118]. Clinical and epidemiological studies have also confirmed that decreased SCD1 is related to decreased risk of breast and pancreatic cancers [119,120,121,122,123].

Studies on the molecular mechanisms underlying the effects of 16:1n-7 on cell proliferation have suggested that the fatty acid possesses mitogenic activity upon exposure of fibroblasts to growth factors [90]. Importantly, the biological effect was not attributable to the free fatty acid itself but to a 16:1n-7-containing inositol phospholipid species that accumulated in the cells upon cell activation [90]. This is fully consistent with recent data demonstrating the accumulation of 16:1n-7 (and also the 16:1n-9 isomer) in selected inositol phospholipid species of activated cells, suggesting novel modes of cell regulation [86]. While this interesting role for 16:1n-7 as a mitogenic signal on its own will require further confirmation and characterization, it adds to the growing notion that individual phospholipid species play specific biological roles in cells [90,91,92,93,124,125,126,127,128].

Recent work is revealing a previously unanticipated plasticity in the lipid metabolism of some tumor types. Instead of relying on the canonical SCD pathway for fatty-acid desaturation, tumor cells appear to exploit a different enzyme, FADS2, and upregulate this alternative route when SCD is inhibited [22]. 16:1n-10, generated by this route, has received attention as a novel marker of cancer cell plasticity owing to the elevated levels of this fatty acid in several cancer cell lines, mouse hepatocellular carcinoma, and primary human liver and lung carcinomas [22,129,130]. Thus, 16:1n-10 biosynthesis through FADS2 constitutes an alternative source of MUFA to support the proliferation of the tumor cells, which may involve, among other actions, increasing membrane fluidity [11,129]. In addition, 16:1n-10 may also affect the EGFR/AKT/mTOR cascade, which is a critical pathway in tumors to support growth, survival, and metastasis [130]. 16:1n-10, however, may not interfere with the mitogen-activated protein kinase signaling cascade [10].

A recent study using stable isotope labeled fatty acids and mass spectrometry measurements in the prostate cancer cell line LNCaP has highlighted the existence of compartmentalized regulation of fatty acid utilization [20]. This may result in disparate metabolic fates and cellular functions for isomeric fatty acids such as those of the hexadecenoic fatty acid family, i.e., 16:1n-7, 16:1n-9, and 16:1n-10 [20]. The differential incorporation of fatty acids into individual phospholipid species raises the intriguing possibility that lipid isomers could be regarded as potential biomarkers for disease progression in tumor tissues, with the hexadecenoic fatty acid family likely playing a prominent role. Figure 5 summarizes the results discussed in this section.

## 6. Conclusions

A MUFA-rich diet has been reported to be beneficial to prevent or ameliorate the symptoms of a number of metabolic diseases. In recent years, 16:1n-7 and its positional isomers, 16:1n-9 and 16:1n-10, have received much attention owing to their anti-inflammatory properties. 16:1n-7, described as a lipid hormone, may exert different effects depending on the organ under study and the disease model. Clearly, more research is necessary to clarify the role of this fatty acid in human health and disease. On the other hand, not much is known yet on the biological significance of 16:1n-9 and 16:1n-10. The former may have utility as a marker of early cardiovascular risk, while the latter may be contemplated as an alternative MUFA source in tumorogenic environments. Future studies should characterize in detail the biological roles of these positional isomers of 16:1n-7 and their influence on metabolic diseases and cancer.

## Data Availability

Not applicable.

## Abbreviations

16:1n-7	palmitoleic acid (*cis*-9-hexadecenoic acid)
16:1n-9	hypogeic acid (*cis*-7-hexadecenoic acid)
16:1n-10	sapienic acid (*cis*-6-hexadecenoic acid)
FADS	fatty acid desaturase
FAHFA	branched fatty acyl esters of hydroxy fatty acids
HFD	high-fat diet
MUFA	monounsaturated fatty acid
NAFLD	non-alcoholic fatty liver disease
NASH	non-alcoholic steatohepatitis
cPLA_2_α	group IVA cytosolic phospholipase A_2_α
iPLA_2_β	group VIA calcium-independent phospholipase A_2_β
SCD	stearoyl-coenzyme A desaturase
